# The Poisonous Principle of Spoiled Corn

**Published:** 1876-12

**Authors:** 


THE POISONOUS PRINCIPLE OF SPOILED CORN.—Prof. C. Lombroso (*Centralbl. f. Med.*, 1876, p. 228) describes two poisonous principles derived from spoiled maize: an oil soluble in alcohol, and an alkaloid. From these may be derived a body closely resembling strychnia, possessing all the chemical and most of the physiological reactions of the latter alkaloid. In frogs, not only tetanic symptoms, but also those of paresis and narcosis, were induced by administration of the oil. In chickens, after prolonged administration of the oil, only paresis and convulsive movements of the head, with inclination to retrograde movements, were induced. The administration to chickens of the alkaloid, on the other hand, induces death in a few minutes, with previous paralysis of the limbs and chronic convulsions. Administered to locusts, fish, mice, etc., the alkaloid gives rise to symptoms similar to those of strychnia poisoning. Prof. L. concludes, therefore, that two distinct poisons are present in spoiled maize.

